# The silent burden of stigmatisation: a qualitative study among Dutch people with a low socioeconomic position

**DOI:** 10.1186/s12889-018-5210-6

**Published:** 2018-04-03

**Authors:** Audrey M. W. Simons, Inge Houkes, Annemarie Koster, Daniëlle A. I. Groffen, Hans Bosma

**Affiliations:** 1 Department of Social Medicine, CAPHRI Care and Public Health Research Institute, Maastricht University, P.O. Box 616, 6200 MD Maastricht, the Netherlands; 2 Department of Data Collection, Statistics Netherlands (CBS), P.O. Box 4481, 6401 CZ Heerlen, the Netherlands

**Keywords:** The Netherlands, Socioeconomic position, Perceived stigmatisation, Self-respect, Qualitative study

## Abstract

**Background:**

In-depth qualitative research into perceived socioeconomic position-related stigmatisation among people living at the lower end of our socioeconomic hierarchy is necessary for getting more insight in the possible downside of living in an increasingly meritocratic and individualistic society.

**Methods:**

Seventeen interviews were conducted among a group of Dutch people with a low socioeconomic position to examine their experiences with stigmatisation, how they coped with it and what they perceived as consequences.

**Results:**

Social reactions perceived by participants related to being inferior, being physically recognisable as a poor person, and being responsible for their own financial problems. Participants with less experience of living in poverty, a heterogeneous social network and greater sense of financial responsibility seemed to be more aware of stigmas than people with long-term experience of poverty, a homogeneous social network and less sense of financial responsibility. Perceived stigmatisation mainly had emotional consequences. To maintain a certain level of self-respect, participants tried to escape from reality, showed their strengths or confronted other people who expressed negative attitudes towards them.

**Conclusion:**

Despite the good intentions of policies to enhance self-reliance, responsibility and active citizenship, these policies and related societal beliefs might affect people at the lower end of our socioeconomic hierarchies by making them feel inferior, ashamed and blamed, especially when they cannot meet societal expectations or when they feel treated disrespectfully, unjustly or unequally by social workers or volunteers of charity organisations.

## Background

Despite all the efforts to reduce the (health) gaps between rich and poor, modern Western societies are still being challenged by socioeconomic inequalities and significant health inequalities [1, 2]. At the same time these societies are increasingly permeated by the belief that economic and social success can be achieved through talent and effort [3, 4]. The downside of this increasingly accepted meritocratic belief in earned success and individual responsibility might be that people at the lower end of Western societies are stigmatised [3–5]; they might be regarded as lazy, incapable or unmotivated [6–9]. Stigmatisation occurs when, within social interactions, a personal attribute is recognised as different (e.g. not wearing the right brand or being unemployed), and the person is devalued because of this attribute. Perceived stigmatisation is the stigmatised person’s subjective experience of this devaluation [10]. In this paper, perceived stigmatisation will refer to perceived devaluation based on attributes related to someone’s socioeconomic position (SEP) (e.g. low income, unemployment, being a social benefit recipient, and/or living in poverty/poor material circumstances).

Previous research has shown that social benefit recipients and people living in poverty often perceive negative judgements and feel degraded, isolated, devalued, blamed and looked down upon [11–17]. We found that over 18% of the general Dutch population perceived some kind of SEP-related stigmatisation, and that people in the lowest income and occupational groups were significantly more likely to perceive stigmatisation (respectively, 22.0% and 27.5%) [18]. However, further in-depth research into perceived SEP-related stigmatisation and its consequences is necessary to understand this possible downside of living in an increasingly meritocratic and individualistic society.

Research into various forms of stigmatisation (e.g. racism) has shown that perceived stigmatisation can have serious physiological and psychological health consequences for the stigmatised individual [19–26]. Some studies found that perceived SEP-related stigmatisation is also associated with poor self-rated health, negative emotions and feelings of inferiority [16, 23]. The consequences of stigmatisation (e.g. for health) depend, inter alia, on how people perceive social situations and how they cope with these perceptions [21, 25, 27, 28]. Different coping strategies for both material deprivation (i.e. poverty) and related stigmatisation were found in a number of studies: overcompensating positive behaviours, violence out of self-protection, not caring about what others think, withdrawing or self-isolating, concealing poverty (e.g. by purchasing expensive items to keep up appearances), and cognitive distancing [27, 29–32]. Sometimes it even seemed that people who experienced long-term poverty appeared to be quite satisfied with the situation (the ‘satisfaction paradox’ [33]). Studies into poverty showed that to protect themselves from the stress related to living on a low income or being unemployed for a long time, people may adapt their standards in order to lessen dissatisfaction and disappointments in life and to become more satisfied with their situation [33, 34].

‘Who is born for a dime, will never be worth a quarter’ was the mainstream idea in Dutch society for a long time. However, in the 1960s the Dutch government developed a generous welfare system and a more egalitarian and easier accessible educational system. As a consequence, inequalities decreased and educational levels rose [35, 36]. Society became more open and individualised, and meritocratic beliefs started to arise: ‘who was born for a dime, could – with some talent and effort – become worth a quarter’ [37–39]. In the 1980s the decrease in inequalities came to an end and increased again [40]. At the same time some changes were made to the welfare system: financial cuts were necessary and free market policies/market competitiveness were introduced, which strengthened the meritocratic way of thinking and increased the focus on self-reliance [41]. A large-scale benefit fraud in the 1990s strengthened the negative beliefs about the (undeserving) poor [42]. In the following years, just as in many other European countries, responsibility for one’s own situation, self-reliance and active citizenship became increasingly important [43, 44]. The strengthened meritocratic beliefs, individualisation of society, and the growing emphasis on own responsibility, self-reliance and participation, all might have strengthened the negative beliefs about people in lower SEP groups. Therefore, the present study aims to provide more insight into the understudied experience of SEP-related stigmatisation from the perspective of low-SEP groups in the Netherlands [18, 45].

Knowing more about the experiences of these people at the lower end of our society might provide tools to enhance the effectiveness of policies (e.g. regarding employment) and professional help to, for example, social benefit recipients or people with financial problems. Since the perception of stigmatisation is a subjective experience, a qualitative study is the most appropriate approach to study perceived stigmatisation in low-SEP groups. Therefore, this qualitative study sought to address the following research questions: (1) What are the experiences of people from lower-SEP groups with SEP-related stigmatisation? (2) How do people from lower-SEP groups cope with SEP-related stigmatisation? and (3) What are the perceived consequences of SEP-related stigmatisation?

## Methods

### Design

A qualitative design with semi-structured individual interviews was used to examine the experiences of SEP-related stigmatisation in a lower-SEP group.

### Participants and context

In 2014, a convenience and purposive sample of people from lower-SEP groups (varying in age, gender and source of income/social benefits) was recruited via a charity organisation that supports poor and often unemployed people by offering easily accessible financial, material or informational support, in a middle-sized city in the southern part of the Netherlands. People in this city are more likely to live on a minimum income than the average Dutch population [46]. People were eligible for this study if they lived on a low income (e.g. had a low-paid job, lived on social benefits or were unemployed), were 18 years or older, and spoke Dutch or the regional dialect. We conducted 16 interviews with 17 persons (one married couple was interviewed). For more information about the sample characteristics, see Table 1.

**Table 1:** Sample characteristics (*n* = 17)

Characteristic	N (%)	
Men	7	(41.2)
Age (range)	32–83 years	
Household type		
Single	11	(64.7)
Single with child(ren)	3	(17.6)
With partner	1	(5.9)
With partner and child(ren)	2	(11.8)
Having a job	–	–
Receiving social benefits	17	(100)
Unemployment Benefit	1	(5.9)
Disability benefit	6	(35.3)
Old-Age Pension	3	(17.6)
Work and Social Assistance	6	(35.3)
Unknown	1	(5.9)
Debt Rehabilitation		
Currently	4	(23.5)
Requested	1	(5.9)
In the past	3	(17.6)
Never	9	(52.9)
Additional support from financial administrator (currently)		
Yes	5	(29.4)
No	12	(70.6)

All interviewees were dependent on social benefits and most of them received disability benefit or work and social assistance. Four people were currently in debt rehabilitation. In the Netherlands, people are eligible for debt rehabilitation when they are no longer able to handle their debts. A personal administrator is then assigned by a court for a certain period to control their finances and help them pay off their debts. After this period, the remaining debts are waived and if necessary people may receive ongoing support from a personal administrator [47].

The life courses of most of our interviewees can be described as ‘hard’: interviewees spoke about growing up in large families or orphanages, being exposed to family problems (e.g. poverty, psychiatric problems, child abuse, divorce and loss of a spouse), earlier debts in adult life and struggles with (psychiatric) illnesses. At the moment of the interviews, none of the interviewees had a job and most of them were long-term unemployed. Jobs of interviewees who had recently worked could be categorised into the lower occupational classes/ working class [48]. Although most interviewees see themselves as ‘poor’ and think to be seen as ‘poor’, we could not judge if they really lived under the poverty line, however they almost all lived in a quite poor financial situation in which they lacked the resources to afford basic necessities (e.g. food, clothes, and health care).

### Procedure and data collection

Participants were recruited by means of posters and flyers with easy-to-read information in the shop and coffee corner of the charity organisation. They received detailed information after signing up for the study, or on request. The interviews took place at a location chosen by the participant (e.g. at the participant’s home or at the charity organisation), and all participants gave their informed consent. The semi-structured interviews were conducted by the first author and lasted on average 70 min (ranging from 25 to 150 min). During the interviews a topic list was used, which was based on the research questions and literature, and included demographics, family composition, living situation, financial situation, social reactions, dealing with social reactions and health. Interviewees also had the opportunity to tell their own story. Topics like stigmatisation and shame were not primed during the interviews. As an incentive, the participants received a shopping voucher of 25 euros. The charity organisation received 250 euros for their collaboration. All interviews were audiotaped and the records were transcribed verbatim. The study protocol was approved by the medical ethics committee azM/UM in Maastricht, the Netherlands (reference number METC 13-4-077, November 2013).

### Analysis

The transcripts were analysed thematically by the first author, using the six phases described by Braun and Clarke [49]. To ensure valid results, the members of the research team participated in a number of peer review sessions, in which several transcripts were read in advance and codes, themes and other important observations were discussed. Analysis was supported by NVivo software.

## Results

**Fig. 1:**
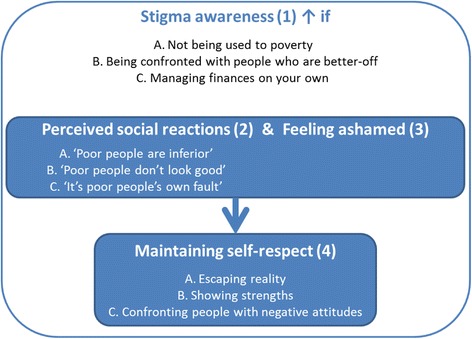
Relation between the themes: Stigma awareness, perceived social reactions, feeling ashamed and maintaining self-respect

Figure 1 shows the four themes that emerged from the data and the relation between the themes: stigma awareness (1) was an overarching theme that influenced the perceived negative social reactions (2) and feelings of shame (3) among the participants, and the ways in which they tried to maintain self-respect (4) when perceiving social reactions or feeling ashamed.

### Stigma awareness

Not all participants seemed equally aware of the existing stigmas related to poverty, unemployment or social benefit recipients or of the stigmatising social reactions they got related to their SEP. Differences in participants’ socioeconomic background (A), composition of their social network (B) and the responsibility they took for their financial management (C) seemed to influence how aware they were of social reactions regarding their financial situation and how they expressed feelings of stigmatisation within their stories.

#### A. Not being used to living on a low income

Interviewees had different socioeconomic backgrounds. About half of the interviewees had grown up in poverty and were used to living on a low income. A number of older interviewees had been born in or just after the Second World War, and grew up in orphanages. Others had never had severe financial problems in the past and had to learn how to cope with these new circumstances. This last group seemed to be more aware of negative social reactions in their environment and the injustice of society, and perceived negative social reactions more often. Interviewees who were more used to living on a low income seemed to have got used to their way of living and, although their stories told us that they were aware of stigmas, they perceived less negative reactions – or were less aware of them – and felt less shame.

*“When I was young we went to the nuns and fathers at the hospital to get food, then you were ‘that kind of a family’ […] You know, I grew up like that and that’s why I fight, I don’t feel ashamed, because I already went through it as a child.”* (A, f68, grew up in poverty)

Besides their socioeconomic background, the participants’ present social network also seemed to influence the degree of awareness of negative social reactions.

#### B. Being confronted with better-off people

Adjusting to living on a low income was also reflected in the social networks of the interviewees. Interviewees who had grown up in poverty seemed to have a more homogeneous network of other people on low incomes, and were less confronted with people who were better off, while interviewees with a more heterogeneous network experienced more confrontations with people who were better off and were more aware of social reactions or feelings of inferiority.

*“And so, like my friends [on higher incomes], they think that I’m a capitalist, because I bought a cupboard for 35 euros, but they don’t know that I then have to live on peanut butter sandwiches for a week. They don’t need to know.” (G, f56)*

For interviewees with a heterogeneous network it also seemed more difficult to feel part of a group; they did not belong to the group of people with no financial worries, but neither to the group of people who were in debt and/or debt rehabilitation.

#### C. Managing finances on one’s own

Living on a low income seemed even harder when interviewees took full responsibility for their own financial management. It was particularly those participants who managed their own payments and savings who mentioned ‘the injustice of society’. Having to struggle with their financial problems on their own – while others got financial, material or informational support – felt unfair, even though they felt proud about managing everything themselves. They sometimes even felt punished for their efforts to prevent debts; they were denied access to additional financial help (e.g. ‘no debts, no problems, no help’) and perceived a lack of understanding, especially from people working at the municipality, local credit bank or Employee Insurance Agency (i.e. social workers, with often a higher professional education in Social work or Social Legal Services) or from volunteers of charity organisations. It was frustrating for interviewees who were free of debts but who were living on a low income without additional support, to see people in debt rehabilitation who received more support (e.g. foodbank, clothes bank, getting things for free or getting interest-free loans) and in the end had more to spend each month than they had. They also felt more stress because of all the bills that had to be paid and the lack of financial support.

*“I think…I mean people who work hard to stay on their feet, to stay out of debt, pay for everything, but live on almost nothing…they are not being noticed.” (G, f56)*

“Y*ou [people in debt rehabilitation] should be glad for all the help you get; I have to do it all myself, and you get so tired of it.”* (R, f52)

Interviewees who had financial assistance seemed to give up part of their own responsibility. They admitted that having financial assistance was sometimes ‘easy’ and relieved them of the monthly payment stress.

“*I’ll go on receiving financial support. You get 85 euros each week and you don’t have to do anything. They pay for things, that’s easy.”* (E, m50)

Thus, participants who took a lot of responsibility and tried to cope with all the financial difficulties themselves were disappointed about the way they were treated by social workers and volunteers.

Interviewees’ socioeconomic background and social network also seemed to play a role in the barrier to giving up financial responsibility and asking for financial support. Participants who had grown up in a more financially stable situation perceived a high barrier to asking for help, and they were more inclined to try to cope with the situation themselves.

*“I don’t want to be dependent, that’s what I fight for, not being dependent. I don’t want that.” (G, f56).*

This overarching theme of differences in stigma awareness also played a more or less important role in the next three themes and subthemes.

### Perceived social reactions

Almost all participants perceived social reactions regarding their financial situation, although in different ways. While some interviewees mentioned negative social reactions explicitly, most of them did not mention negative reactions or perceived stigmas directly, but expressed for example how hard they always worked. This could indicate that they were aware of stigmas relating to poor people or people on social benefits, for example about ‘the poor being lazy’, but were less aware of the social reactions based on these stigmas. As described in the first overarching theme, this could depend on peoples’ experiences with poverty or living on a low income. Perceived social reactions were categorised into three subthemes:

#### A. ‘Poor people are inferior’

In contacts with people in society (e.g. neighbours or people in the street), social workers and volunteers of charity organisations (e.g. at the foodbank), participants experienced reactions or treatments that made them feel they were worth less than others because of their financial situation – sometimes even less than others who were also living on a low income – and they felt looked down upon. It was particularly those who were struggling with living on a low income, taking responsibility for their own financial affairs and without receiving additional financial support (e.g. special benefits), but were able to avoid debts, who experienced unequal treatment at the foodbank, the second-hand shop of a charity organisation or the municipal authorities.

*“…that month that I went [to the foodbank], you go along the tables and they put food on it and I get a carton of yogurt and a carton of pudding from the ‘Aldi’ [cheap supermarket] while someone else gets, for example, 3 or 4 cartons of ‘Mona’ pudding [premium brand]…Why? Why don’t they distribute it fairly?*” *(M, f53)*

Interviewees felt looked down on in social situations, but could not always mention why or what was happening at the moment. It was more like a feeling, without something actually happening.

*“When you visit people with nice pearl necklaces, you feel that…without them talking about it, you feel it, I at least do. I feel it…” (G, f56)*

For some participants the ‘status’ of their neighbourhood was important; living in a ‘good’ neighbourhood made them feel better because people saw them differently, more favourably.

*“People do ask ‘where do you live?’ but simultaneously they think they can tell you where you live. Telling where you come from can have a different impact, in conversations or at work for example. I notice that. […] Yes, people give you different ‘looks’. Yes different…pleasant.” (D, f57, living in a ‘good’ neighbourhood)*

Sometimes, interviewees also felt that others believe they did not deserve luxury or nice things because of their financial situation, although they themselves think they did, especially because of all the personal and financial struggles.

*“I got this apartment, and I took over the tiled floor and the washing machine, so I got a real nice apartment, but the people in the hallway they’re jealous, but I can’t help I’m the lucky one, but then I think ‘I’ve lived in a shelter for 4 years, so what’s wrong with this?” (R, f52).*

In addition to direct experiences of negative social reactions regarding inferiority, participants often anticipated and feared getting negative reactions. The same is true for the next subtheme.

#### B. ‘Poor people don’t look good’

Interviewees expressed how they took care of their appearance (e.g. clothes) to avoid negative social reactions, to fit in with peers, or to feel better about themselves.

*“In the evening, I washed my clothes and then I put them on again the next day […] nobody would notice, as long as they were clean and I showered” (A, f68).*

Some expressed a lack of interest in wearing appropriate or expensive clothes themselves: they would rather use the money for other things, like clothes for their children to prevent bullying or going out with friends, or they argued that their lack of interest in wearing appropriate clothes was not because of their financial situation but because they found new clothes unnecessary.

*“I can wear the same trousers for 2 years, I don’t care. But it’s different for my children, they go to school and might be bullied you know.” (L, f?)*

“*I don’t care [about clothes]… I always think ‘you guys, go ahead and spend your money on them…I can’t, but I have enough clothes, I don’t need new clothes every two months, that’s not necessary’ [..] I just wear my work clothes.” (O, m60).*

Interviewees also proudly showed or told how good their homes looked, even with second-hand furniture.

“*I furnished my whole house with stuff from [the shop of the charity organisation] and from second-hand shops and I furnished it very nicely.” (C, m61)*

For some it was frustrating and sometimes shameful that they could not afford furniture, wallpaper, paint, or decorations to make their house into a home, but they put their circumstances into perspective and said they were glad to have place of their own.

*“I had a nice house, you know, with everything, television, bedrooms…a widescreen TV on the wall and now I’m watching a small one. It doesn’t hurt, because I saw the other side of the medal too […] I’m thankful for having a roof over my head and I eat and drink every day.” (B, m51)*

Participants’ stories showed that their financial situation and the awareness of the necessity to keep up appearances was often a difficult combination, as they wanted to take care of their appearance, but did not always have the financial means to do so. The participants’ background or social network seemed to play a less important role in these experiences than in those regarding inferiority; they all seemed to be aware of the necessity of trying to look presentable.

#### C. ‘It’s poor people’s own fault’

Participants mentioned negative reactions regarding their unemployment. Some emphasised how hard they were working at the moment (e.g. as a volunteer) and how active they were, or they wanted to show how hard they had always worked by listing all the jobs they had in the past. Or they emphasised that their current situation (e.g. unemployment) was not their fault (e.g. not because of laziness or not being willing to work).

“*I’m always busy. I worked before, because I took every job that I could, I even worked in industry, at a fast food restaurant, in shifts, I worked there for a few months. I also worked for a farmer harvesting asparagus and a little bit in the catering industry. […] Because I’m always busy, I won’t get comments like ‘they’re always sitting around doing nothing’.”* (D, f57)

Participants often felt blamed and felt the pressure to justify why they were not in work; although they wanted to work, they often felt unable to work because of health problems or family commitments.

*“I’ve been on benefits since 2007, first my children were small and I had to cope with a lot myself because of my problems in the past. Then I got two children and I was unable to work, that’s how it went.” (L, f?)*

Another group of interviewees seemed to show the fear of being blamed by ascribing the cause of their financial problems and lack of work to bosses, the government, low benefits, the euro or foreigners taking their jobs.

“*The euro, health insurance and the taxman cause problems for a lot of people, not only for me […] It was not my fault that I got into debt, that’s the worst part of it. If I had just gone on holiday, bought cars, etc., then you know why you’re in debt, but I got into debt another way…and that’s difficult to say.” (K, f36)*

Some interviewees also expressed their negative attitude towards working (for money or for a boss), especially after some negative experiences with former employers.

*“I will never work for the high and mighty in the Netherlands again. This is the third employer here in the Netherlands who has fired me, even though I also put in 100% effort. […] I have to apply for a job four times a month… and I do that! I apply, send off the applications, done…but I won’t work anymore.” (B, m51)*

Interviewees also experienced social reactions that gave them the feeling that they were responsible for their own financial situation, or they perceived a lack of understanding of their financial hardship and the support they were receiving.

“*My daughter was ill […] she didn’t work, so yeah I paid for her medication, I paid her rent and all other expenses, so my savings were gone…then the people at the municipality [when applying for additional social benefits] told me ‘You shouldn’t have done that’. I shouldn’t have helped my daughter, I should have saved up the money.” (M, f53)*

Although most of the participants described experiences of or the fear of experiencing negative social reactions regarding their own responsibility, those with long-term experience of living on a low income or being unemployed seemed to be more likely to blame others for their situation.

### Feeling ashamed

Participants’ feelings of shame also seemed to differ with their background and the composition of their social network; those with long-term experience of living on a low income and those with a more socially homogeneous network (in terms of SEP) expressed less shame.

Shame was reported to occur in various situations: for example when remembering their successful past:

*“Shame…yes… think of it this way…I always worked on my career [being a successful DJ] […] …then going down [in income/status], while people know you had bags of money in the past.” (E, m50).*

Or when others uncovered their previous or current poor financial situation:

*“On television they [the interviewers] would just say ‘O I heard you also used to beg in the street?” (A, f68).*

Or when they were unable to pay for gasoline, when people saw their homes or when they could not give something in return after being given something.

*“I used to live in a very dirty apartment, I had no money to do it up [for the film crew]. I got a stand from the second-hand shop, put a vase from the second-hand shop on top, with roses in it that I couldn’t really afford […] bought a poster. And I said ‘would you please film me in that corner there, cause I don’t have the money to do it up. I’ve only just moved in here. Would you please film that part?’” (A, f68, who was interviewed for TV)*

It was also embarrassing when they had to tell their story over and over again.

*“…you have to tell your story again and again and explain what’s the matter […]. But after a while it stops you from doing things because you get fed up, you’re tired of telling your story once again, explain your situation again, as you’re seen as a beggar, you’re just begging in fact.” (R, f52)*

Some also expressed that they did not feel ashamed or that it was not necessary to feel ashamed about their financial situation, because they were used to it, did not care, said they were lucky with all the help, or because it was not really a problem since many people had debts.

*“… I don’t care about that [wearing second-hand clothes], I’m not ashamed about it. There are people who feel ashamed about that. But I’m not.” (H, f82)*

### Maintaining self-respect

Having to tell their story over and over again to justify why they need help, losing their autonomy in life because of their limited resources and dependence on others, being deprived of privacy, and being treated unequally or without respect appeared to affect interviewees’ self-respect. This was most noticeable in the strategies interviewees used to cope with social reactions; maintaining self-respect appeared to be the main goal of their strategies. Their strategies seemed to differ with participants’ backgrounds and social network. For example, those who were more used to living on a low income and who had a more homogeneous social network (in terms of SEP) seemed to be more likely to cope by denying, playing tough and attaching less value to certain aspects of life, whereas those who were not used to living on a low income and had a more heterogeneous network were more likely to try to conceal their situation and maintain their self-respect by showing how proud they were of their achievements and emphasising their positive characteristics. They were also more likely to actively confront people who show negative attitudes. The coping strategies could be roughly categorised into three subthemes:

#### A. Escaping reality

Interviewees tried to deny or conceal their difficult situation, to themselves or to others; they would conceal their financial problems to family and friends, deny perceiving negative social reactions and attach less value to status symbols to protect themselves from feeling bad.

##### Concealing financial problems

Most interviewees tried to conceal their financial problems by taking care of their appearance and behaviour in public, by living in a better neighbourhood, or by just not telling family members and friends about their financial problems.

*“I never really talk about my, err, finances, you know. Because, well, I want to feel like I still have a bit of pride about myself, you know.” (G, f56).*

They also tried to hide their situation by looking for excuses, by staying at home and avoiding social contact, or by avoiding shops.

*“It regularly happens that we say ‘we don’t feel like it’ even though we really would like to do it, but we can’t.” (P, m50).*

##### Denial of negative social reactions

Some interviewees denied perceiving negative reactions regarding their financial situation when they were asked about negative social reactions. However, indirectly, and perhaps also unconsciously, they mentioned the need to look good or appropriate or blamed others for their financial situation. For example, one man when asked if he got any reactions regarding his living situation, since he was living on small income in a quite high SEP neighbourhood, denied this. However, a few seconds later he told about a conflict with his neighbour who had complained about him not taking care of his animals:

*“No, not really. […] You don’t come into contact with people there [in his neighbourhood]. […] And I had a cat and a dog, and then the woman next doors started to feed my cat. And at a certain moment I got a note through the letterbox saying I should feed my cat better. […] I got angry and I didn’t speak to her again.” (C, m61).*

##### Attaching less value to SEP indicators

During the interviews some participants expressed a negative attitude towards working (again) or money, and denied the importance of a high educational level.

*“Yeah, she was advised to take HAVO (a higher level of secondary school) but that was doubtful, so she went for MAVO (a lower level). She knowns what she wants to be in the future anyway. […] When you take HAVO, that means you can go into HBO (a higher level of tertiary education), which would be nice, but MBO (a lower level, accessible with a MAVO diploma) will also get her there.” (K, f36).*

They seemed to attach less value to aspects in life that are important for a high position in society.

#### B. Showing strengths

By playing tough, emphasising positive characteristics, showing pride, and collecting objects, participants seemed to cope with the lack of status symbols like money or big cars.

##### Playing tough

A few male interviewees did not mention feelings of inferiority directly, but their behaviour during the interviews seemed to show otherwise; they showed ‘macho behaviour’, aggression or emphasised how strong, self-confident or smart they were.

*“Then my employer fired me and I ran away to avoid killing him, […] I had already come across him once before, when he was a bit drunk and I looked for a piece of wood … and struck at him, but fortunately I didn’t hit him.” (B, m51)*

By ‘playing tough’ they seemed to protect their self-respect from being damaged by others.

##### Emphasising positive characteristics and showing pride

Interviewees also seemed to try to maintain their self-respect by emphasising their positive characteristics (e.g. being loved, sociable and empathic).

*“I know lots of people and they all love me. So that’s nice, right?” (Q, f73)*

Some were proud of their achievements (past or present), of how they handled their difficult financial situation, and of how they furnished their homes with things they found, bought in second-hand shops, or were given by others.

*“Honestly, can’t I be a bit proud of the fact that I have avoided that [i.e. getting into debt]” (G, f56)*

A number of interviewees also proudly told about or showed their collections of music (e.g. CDs or DJ equipment), books, statues and figurines, or pets. And although it sometimes cost them a lot of money, their collections or pets meant a lot to them. These material things seemed very important to them, especially when they had only few possessions.

*“You might get rid of it, but then somebody else will get the credits. So I’m not gonna do that.” (E, m50, about his DJ/music collection)*

Emphasising positive characteristics and showing pride made interviewees feel good about themselves and seemed to improved their self-respect.

#### C. Confronting people who show negative attitudes

Besides trying to escape reality or trying to maintain self-respect by showing their strengths, some interviewees also tried to confront others regarding their negative social reactions or negative attitudes. One man challenged people to be more specific regarding statements they made or opinions they expressed.

*“‘Just tell me where?’ if they say ‘There’re enough jobs!’ ‘Well then, just give me an address and I’ll go there.’ ‘Well, I can’t.’ So well, there’s no work then, is there?!” (P, m50).*

One woman wanted to start a radio program to give homeless people a voice and enable them to share their experiences and stories, with the intention of opening up listeners’ eyes.

*“So people will understand if they see somebody walking by, that it’s not just …” (R, f52*).

She even went to the town council to talk with politicians about the unequal treatment at the foodbanks.

*“I can’t stand injustice. […] I’m very quiet and calm, but when I see injustice, […] I get angry. […] I went to the town council and I know this woman and then I tell her about it, hoping she’ll do something about it [i.e. injustice at the foodbank].”(R, f52)*

Another woman wanted to go on participating in society so she could show that people who were unemployed were not lazy or unwilling to work and prevent negative reactions.

*“No, not as such [about social reactions to her unemployment], but that’s because I’m always busy, you know, so I don’t get these comments like ‘Well, they’re sitting around all day doing nothing’.” (D, f57).*

Confronting people regarding their attitudes or beliefs seemed to be an effective way of coping for some interviewees; however, it also seemed hard for participants to confront people, especially when social reactions were not expressed very directly.

## Discussion

The aim of this study was to examine in greater depth the experiences of SEP-related stigmatisation in Dutch social benefit recipients. From the 17 individual semi-structured interviews, four themes emerged: (1) awareness of stigma, (2) perceived social reactions, (3) feeling ashamed, and (4) maintaining self-respect. These themes enabled us to answer our research questions: (1) What are the experiences of people from lower-SEP groups with SEP-related stigmatisation? (2) How do people from lower-SEP groups cope with SEP-related stigmatisation? and (3) What are the perceived consequences of SEP-related stigmatisation?

Our study showed that people at the lower end of the social hierarchy in the Netherlands feel stigmatised because of their SEP. Participants perceived the stigma of being inferior, being physically recognisable as a poor person, and being responsible for their own financial problems. Participants often talked (directly or indirectly) about perceived or anticipated negative social reactions regarding their SEP, or they used compensation strategies to cope with (real or anticipated) negative reactions and to maintain their self-respect. Similar results were found in recent studies by Kampen, Elshout and Tonkens [39, 50], who found that long-term unemployed Dutch people struggled with self-esteem and self-respect, and that they also felt inferior, looked down upon and ashamed.

Our interviewees differed in their awareness of negative reactions regarding their SEP. This is also called stigma consciousness: they might all be aware of their stigmatised status, however people might focus more or less on their stigmatised status and expect more or less stigmatising social reactions [51]. Participants seemed more aware if they had had less experience of poverty or living on a low income in the past, had a heterogeneous social network (in terms of SEP) and tried to manage their own finances. Injustice in society was particularly felt by participants who wanted to be independent and took responsibility for their own finances. They felt that their efforts were not being recognised and that they were treated unequally by social workers and volunteers of charity organisations, particularly compared to other people who lived on a low income but who did not take responsibility for their own financial matters. Participants who had experienced poverty or living on a low income in the past and those with homogeneous social network (in terms of SEP) seemed to be more used to living on a low income. The ‘satisfaction paradox’ can explain why we found less stigma awareness among participants with long-term experience of living on a low income. Learned helplessness, low control beliefs and the cognitive dissonance of wanting a more satisfying life but being unmotivated because of disappointments in prior unsuccessful attempts can explain this satisfaction paradox in this group. [33, 34, 52]. When people become less active and lose motivation (e.g. not looking for work, not taking responsibility for their financial management) because they found out that their efforts had no effect, they may adjust to their situation by resolving the cognitive dissonance through lowering their standards, and this lowering of standards could result in less stigma awareness. On the other hand, when people see ‘satisfied poor people’ in society this might also enhance stigmatisation, as their circumstances might be perceived as being their own choice (e.g. not being in work or living in poverty) [33]. Another issue that could make participants less aware of negative social reactions regarding their socioeconomic situation could be the high prevalence of other problems in the family. Most interviewees’ families could be categorised as multi-problem families; they were struggling not only with financial problems but also with severe health problems, psychiatric problems, behavioural problems of family members, children being taken into care, or tensions between family members. It is possible that they were more aware of negative social reactions (stigmas) regarding these other problems (e.g. being bad parents or psychiatric patients) than of reactions regarding their financial situation) [16].

The goal of participants’ strategies to cope with classism was to maintain some level of self-respect, which could have been damaged by living at the lower end of the social hierarchy in a meritocratic society. Swierstra and Tonkens [4] and Elshout [39] described a number of ways of maintaining self-respect in a world where self-respect of people in lower positions is undermined because it is believed that they deserve their low SEP and have to be ashamed of themselves. We recognised some of them in our study: criticising ideology (e.g. blaming others for not having equal chances in life), shortcuts and changing the rules (e.g. stealing to earn respect and to have money), and refusing to take part in the competition (e.g. no longer being willing to work). The coping strategies found in our study are consistent with strategies found in previous research into coping with poverty and related stigmatisation, for example compensating behaviour like aggression, denial and concealing and withdrawing or self-isolating [27, 29–32]. The ‘satisfaction paradox’ can also explain some of the coping strategies we found in participants with long-term experience of poverty, for example, attaching less value to certain aspects of life resembles lowering one’s standards in life, making a disadvantaged life easier to accept.

In our study the consequences of perceived SEP-related stigmatisation were not directly mentioned. Perceived stigmatisation affected emotions (e.g. anger, frustration, shame, stress etc.) and self-respect, but they did not mention consequences to physical health. When physical health consequences were mentioned, they were often related to stress, lifestyle and their financial situation (e.g. smoking because of stress, not being able to buy fruits and vegetables or to pay for necessary healthcare). Nevertheless, it is known that emotions like anger and frustration, feelings of stress and lack of social participation can also negatively affect people’s physical health [19, 24]. Participants’ interpretations of social situations and their coping strategies determined how stressful a situation was to them. This might also explain the role of the participants’ socioeconomic background: people with long-term experience of poverty may have learned better how to cope with stigmatisation in order to reduce the stressful consequences [28, 33, 34].

During the interviews it also became obvious that participants compared their situation and the way they were treated with people in similar circumstances. Participants felt frustration and injustice when they were treated unequally by social workers or volunteers of charity organisations. There was a strong focus on who gets what and how much (e.g. food from the foodbank or additional benefits from the government). These observations were consistent with De Botton’s theory of status anxiety, which focuses on comparisons between close peers instead of people who are more different [3].

SEP-related stigmatisation is also known as classism: the marginalisation of those who are perceived to be in a different social class [53]. However, we chose not to use this term in this study because it refers especially to stigmatisation and discrimination based on ‘class’, although it is used in a broader sense in other studies [54, 55]. In this study, we did not focus on class as the most important SEP indicator. Interviewees’ experiences with stigmatisation were related to different indicators of SEP [56]: being unemployed, receiving social benefits, being poor, living in poor material circumstances, having an inferior status in society. Further, people often do not know why they are stigmatised [16]. Moreover, to experience stigmatisation, their stigmatised identity has to be disclosed: becoming visible to or known by others (e.g. living in poor material circumstances, visiting the food bank or being unemployed) [57]. Only looking at ‘class’ would narrow our view on the experiences with stigmatisation too much.

### Implications

Although interest in and awareness of the struggles characterising the daily lives of people living in (long-term) poverty or unemployment is rising [58], this might be one of the first scientific studies into perceived SEP-related stigmatisation in the Netherlands. We discussed what our results meant for society, however, our ideas about the implementation need further support by additional studies into SEP-related stigmatisation in the Netherlands (e.g. a study into the prevailing SEP-related stigmas in the Dutch society).

Just as in many other Western societies, there has been increasing emphasis on individualisation and self-reliance in Dutch society in recent years; people are expected to take responsibility for themselves and to participate in society [44], These expectations will increase the stigmatisation of people who are unable to take full responsibility in this respect [39]. As our participants’ stories showed, the lack of money or work is not the only problem in their lives, and the pressure to work, participate and take responsibility will not lead to a solution for their situation: it will in fact increase participants’ struggles with feelings of inferiority and blame. Policies aiming at activating citizens and enhancing self-reliance have good intentions, but can also create collateral damage in the already difficult lives of people at the lower end of our socioeconomic hierarchy; they might feel treated disrespectfully, unjustly or unequally by social workers who base their approach on policies and societal beliefs [39, 59]. Finding a balance between helping and impairing the situation of people at the lower end of our socioeconomic hierarchy in Western societies requires thinking about ways to motivate them to participate and take responsibility while simultaneously ensuring a respectful, just and fair approach and avoiding stigmatisation [59]. Thereby, social workers and volunteers working in charity organisations should also be aware and act upon varying abilities of people in taking own responsibility and to comply with regulations and agreements; financial problems are often not the only problem they face and failures to comply may be due to more than a lack of motivation or ability [59].

This study also underlines the need for further research into perceived SEP-related stigmatisation as a possible explanation for the hard-to-change socioeconomic health inequalities in modern Western societies. When our beliefs affect how we as society, including social workers and other professionals, approach people at the lower end of the socioeconomic hierarchy, interventions aimed at reducing the health gap might be unsuccessful or hurt people in low-SEP groups even more, especially when interventions (unintentionally) enhance stigmatisation. SEP-related stigmatisation might be a refractory problem in Western societies, but creating awareness of our (unconscious) stigmatising beliefs and its consequences will be the first step towards changing how we think about and approach this vulnerable group [60].

### Methodological reflections

The strength of this study was the interviewing method. Even though there was a topic list, participants had the opportunity to tell their story, which provided rich data. Perceived SEP-related stigmatisation was not primed in the interviews, in order to prevent socially desirable answers or fierce denial.

This study had some limitations as well. First, due to our open method of interviewing we did not gather detailed information on life course and employment history of the interviewees (as this was not the main research question). It is important to collect this information in future research to be able to relate their experiences with stigmatisation and their background more accurately. Second, since most of the participants were also clients of the charity organisation–four participants were only recruited via the charity organisation but not clients–they may already have broken through a barrier of shame. We assume that experiences of SEP-related stigmatisation might be even worse in low-SEP groups which are not visible to charity organisations, because they might avoid charity organisations due to perceived stigma and shame. This group might be the working poor [61]; over 40% of people living in poverty in the Netherlands have a paid (part-time) job or are self-employed [62], however they make less use of financial support offered by the government [61, 63] . Because we missed this group, we cannot generalise our results to all low SEP groups. Third, since this study was conducted in the Netherlands, our results cannot be simply generalised to other countries. However, trends of individualisation and a focus on self-reliance are found in many Western societies [43, 44], so the stories of our participants could well resemble those found in other parts of Europe. Fourth, as we found during the interviews, only a few participants told us directly or without priming about the negative social reactions they had encountered. During the analysis we were aware of the risk of our subjective interpretation of the participants’ stories; our backgrounds and values as researchers may have been quite different from those of our participants and may have led to biased interpretation of the data. However, we tried to minimise our subjectivity by discussing the interviews in our research team (i.e. peer review).

## Conclusion

On top of all the daily struggles of living on a low income with severe family problems, our participants had to deal with the experience of SEP-related stigmatisation. Almost all participants perceived social reactions related to being inferior, being physically recognisable as a poor person, and being responsible for their own financial problems. They also experienced feelings of shame. The awareness of SEP-related stigmatisation differed with participants’ SEP background, social network and sense of responsibility. Maintaining self-respect in an increasingly meritocratic society was an important goal for them when dealing with SEP-related stigmatisation. It will be important to remember that, despite the good intentions, policies enhancing self-reliance, everyone’s own responsibility and active citizenship can affect people at the lower end of our socioeconomic hierarchy by making them feel inferior, ashamed and blamed. This will be even worse in situations where they cannot meet the high societal expectations or when they feel treated disrespectfully, unjustly or unequally by social workers and charity organisations.

## Abbreviations

HAVO: A higher level of secondary school
HBO: A higher level of tertiary education
MAVO: A lower level of secondary school
MBO: A lower level of tertiary education
SEP: Socioeconomic position
